# Supplementary material for: Variation in pathogen load and the pathogen load–infectiousness relationship broaden avian malaria’s distribution
Source: Nat Commun. 2026 Feb 10;17:1213. doi: 10.1038/s41467-026-68927-x (PMC12891551; doi:10.1038/s41467-026-68927-x)
Supplement: Supplementary file 1 — Supplementary Information [file 41467_2026_68927_MOESM1_ESM.pdf]

Supplementary Information

Variation in pathogen load and the pathogen load–infectiousness relationship broaden avian malaria's distribution

Christa M. Seidl[1*], Katy L. Parise[2], Isaiah Ipsaro[1], Sarah Leach[1†], Delson Hays[1†], Ranger Morimoto[1†], Kelsey Banister[2], Francisco C. Ferreria[3], Elizabeth Abraham[4], Jeffrey T. Foster[2], Eben H. Paxton[4], A. Marm Kilpatrick[1]

1 Department of Ecology and Evolutionary Biology, University of California, Santa Cruz, CA, 95060

2 Pathogen and Microbiome Institute, Northern Arizona University, Flagstaff, AZ, 86011

3 Center for Vector Biology, Rutgers University, New Brunswick, NJ, 08901

4 U.S. Geological Survey, Pacific Island Ecosystems Research Center, Hawaiʻi National Park, HI, 96718

[†]These authors contributed equally: Sarah Leach, Delson Hays, Ranger Morimoto

<u>Current Affiliation</u>:
Christa M. Seidl: Maui Forest Bird Recovery Project, Pacific Cooperative Studies Unit, University of Hawaiʻi at Mānoa, Makawao, HI, 96768

***Corresponding Authors:** seidlcm@gmail.com

This file includes:

- Supplemental Methods
- Tables S1 to S5
- Figures S1 to S11
- Supplemental Information References

This document contains Supplemental Text, Tables and Figures. The supplemental text describes the simulations used to examine two methods for estimating the contribution of species to transmission. The supplemental figures and tables include the geographic locations for the sites where bird communities were surveyed by point counts, the sites where the *Plasmodium relictum* isolates that were used in experimental infection studies were collected, and the collection locations for *Culex quinquefasciatus* egg rafts, an image of our mosquito feeding cylinder in use, the outputs for our generalized linear mixed effects models, which used the glmmTMB package[1] in R Version 4.4.1 (2024-06-14)[2], and additional data figures from our experimental and field work.

Supplemental Methods

Simulations comparing the use of relative infection prevalence and directly measured mosquito relative host utilization for estimating species contributions to transmission

We performed simulations to assess the accuracy of two methods for quantifying the contribution of species to transmission. The two methods were: 1) using relative infection prevalence to estimate host utilization (as described in the methods), and 2) measuring mosquito relative host utilization by identifying host DNA in blood-fed (engorged) mosquitoes and measuring host abundance[3,4]. As described in the main text Methods, the relative contribution of each bird species to R_0 is: $Rel.Contribution\ to\ R_0 = \frac{a_j^2}{n_j}\frac{s_j c_j}{(\mu_j+\gamma_j+\alpha_j)} = f_j^2 n_j s_j c_j d_j$

Where f_j is the host utilization (sometimes called feeding preference or forage ratio) ($f_j = a_j/n_j$) for species *j*, a_j is the fraction of blood meals from host *j*, n_j is relative abundance, s_j is susceptibility to infection, c_j is average infectiousness, and d_j is the average duration of infectiousness. We compared the two methods listed above for estimating host utilization values and the relative contributions to transmission. We simulated unmeasured variation in susceptibility (s_j ~U(0.8, 1)), the relative duration of detectable infection/infectiousness (d_j ~U(0.5,1)), and host utilization (f_j~Lognormal(0,0.3)). We fixed c_i and n_j to focus on the impact of different methods to estimate host utilization, since both parameters are measured in both methods and wouldn't contribute to deviations from true contributions to transmission (but both

influence the contribution of species to transmission). We then calculated the true contribution of each of N = 30 species to transmission using all parameter values, and compared this to the contribution to transmission using either host utilization, f_j, estimated directly, or host utilization estimated using relative infection prevalence, as described in the main text Methods (Fig. S11). Note that in both cases we assumed variation in susceptibility, s_j, and the duration of infectiousness, d_j were unmeasured and not used in the calculation.

Supplemental Tables

Table S1. Results from a generalized linear model with a binomial distribution and a logit link with the prevalence of disseminated avian malaria infections (Plasmodium DNA detected in head/thorax/legs combined) in *Culex quinquefasciatus* as the response variable and Bird Ct score, Temperature and Days since feeding and two two-way interactions, plus age and study year (a categorical predictor that accounts for differences in mosquito populations used) as predictors. Cox-Snell pseudo-R^2 = 46%.

**Predictor**	**Coef.**	**SE**	**Z**	**P-value**
**Intercept**	-14.25	4.81	-2.96	0.0031
**Bird Ct Score**	0.35	0.17	2.07	0.039
**Temperature**	0.61	0.23	2.67	0.0076
**Days since feeding**	-0.10	0.080	-1.24	0.22
**Mosquito age**	0.038	0.014	2.77	0.0056
**Yr: 2022**	-0.35	0.27	-1.30	0.19
**Yr:2023**	0.94	0.43	2.19	0.029
**Bird Ct Score* Temperature**	-0.018	0.0077	-2.36	0.018
**Temperature*Days since feeding**	0.0081	0.0042	1.96	0.05

Table S2. Results from a generalized linear model with a binomial distribution and a logit link with the prevalence of avian malaria infections in *Culex quinquefasciatus* abdomens as the response variable and Bird Ct score, Temperature, Mosquito age, study year (a categorical predictor that accounts for differences in mosquito populations used) and Days since feeding and a two-way interaction between Bird Ct score, Temperature as predictors. The two-way interactions between Bird Ct score and Days since feeding and Temperature and Days since feeding were not significant (P = 0.35 and P = 0.40 respectively) and were not included. Cox-Snell pseudo-R^2 = 42%.

**Predictor**	**Coeff.**	**SE**	**Z**	**P-value**
**Intercept**	-13.90	5.84	-2.38	0.017
**Bird Ct Score**	0.56	0.20	2.80	0.0052
**Temperature**	0.92	0.25	3.61	0.00031
**Days since feeding**	0.033	0.015	2.23	0.026
**Mosquito age**	-0.046	0.015	-3.09	0.0020
**Yr: 2022**	-1.19	0.32	-3.72	0.00020
**Bird Ct Score* Temperature**	-0.032	0.0088	-3.63	0.00029

Table S3. Point count survey locations for Hawaiʻi Island in 2019-2020. Latitude/Longitude location is the center of a 4-hectare (200 m × 200 m) area. Species relative abundances used in Fig. 3 are the sum of the total observations of a species divided by the total of all species observed over four sequential 6-min surveys conducted 200 m apart.

Site Name	Site Code	Forest Description	Latitude	Longitude	Elevation (m)	Survey Date
Iwalani Hilo, HI	IWAL	Suburban, city	19.6858	-155.0778	29	4/23/20
Hawaiʻi Paradise Park Site 1, HI	MARC	Agricultural	19.5777	-154.9659	60	5/9/19
Kapoho, HI	KAPO	Agricultural	19.4985	-154.8537	62	4/16/20
Hawaiʻi Paradise Park Site 2, HI	CASH	Agricultural	19.5682	-154.9724	77	5/9/19
Nanawale Forest Reserve, HI	NANA	Wet ohia forest	19.5373	-154.9032	100	3/16/20
Malamalama Waldorf School, HI	MALA	Agricultural	19.5586	-154.9728	105	5/9/19
Keauʻohana Forest Reserve, HI	KEAU	Wet ohia forest	19.4188	-154.9524	250	4/15/20
Manuka State Park, HI	MANU	Dry ohia forest	19.1105	-155.8246	600	5/11/19
Ainahou Ranch Unit, Hawaiʻi Volcanoes National Park, HI	AINA	Wet ohia forest	19.3436	-155.2305	921	3/11/20
Upper Waiakea Forest Reserve, HI	UPWA	Wet ohia forest	19.5672	-155.2306	1100	1/30/20
Kahuku Ranch Unit, Hawaiʻi Volcanoes National Park, HI	KAHU	Dry ohia forest	19.1336	-155.7005	1270	5/23/19

Table S4. Relative importance of high and low parasitemia phases of infection for Hawai'i 'Amakihi (*Chlorodrepanis virens*) and 'Apapane (*Himatione sanguinea*) for infecting mosquitoes with *Plasmodium relictum*. The number of infectious mosquitoes generated assumes one mosquito fed on each species every day following infection for the duration of the high and low parasitemia phases, with a probability of disseminated infection 10 d after feeding at 24 °C (Fig. 2).

**Species:**	***C. virens***		***H. sanguinea***	
**Quantity**	**High phase**	**Low phase**	**High phase**	**Low phase**
Duration (d)	38	961	60	789
Mean infectiousness	41.6%	22.7%	42.0%	27.2%
# infectious mosquitoes	19.4	217.8	25.2	215.0
Ratio of # infectious mosquitoes (Low/High)	217.8/19.4 = 13.8		215.0/25.2 = 8.54	

Table S5. Species observed in point count surveys from locations on Hawai‘i Island in 2019-2020. Observed at sites in Table S3 & Fig. 4. Starred(*) species appear in Fig. 4.

Common Name	Genus	Species	Family
African Silverbill*	*Lonchura*	*cantans*	Estrildidae
‘Apapane*	*Himatione*	*sanguinea*	Fringillidae
Cattle Egret	*Bubulcus*	*ibis*	Ardeidae
Domestic Chicken*	*Gallus*	*gallus*	Phasianidae
Common Myna*	*Acridotheres*	*tristis*	Sturnidae
Common Waxbill*	*Estrilda*	*astrild*	Estrildidae
Erckel's Francolin	*Pternistis*	*erckelii*	Phasianidae
Hawai‘i ‘Amakihi*	*Chlorodrepanis*	*virens*	Fringillidae
Hawai‘i ‘Elepaio	*Chasiempis*	*sandwichensis*	Monarchidae
Io (Hawaiian hawk)	*Buteo*	*solitarius*	Accipitridae
House Finch*	*Haemorhous*	*mexicanus*	Fringillidae
House Sparrow*	*Passer*	*domesticus*	Passeridae
Chinese Hwamei*	*Garrulax*	*canorus*	Leiothrichidae
Japanese Bush Warbler*	*Horornis*	*diphone*	Cettiidae
Java sparrow	*Lonchura*	*oryzivora*	Estrildidae
Kalij pheasant	*Lophura*	*leucomelanos*	Phasianidae
Northern cardinal*	*Cardinalis*	*cardinalis*	Cardinalidae
‘Ōma‘o*	*Myadestes*	*obscurus*	Turdidae
Red-billed leothrix*	*Leiothrix*	*lutea*	Leiothrichidae
Red-crested cardinal	*Paroaria*	*coronata*	Thraupidae
Saffron Finch*	*Sicalis*	*flaveola*	Thraupidae
Scaly-breasted Munia*	*Lonchura*	*punctulata*	Estrildidae
Spotted Dove*	*Spilopelia*	*chinensis*	Columbidae
Warbling White-eye*	*Zosterops*	*japonicus*	Zosteropidae
Yellow-billed Cardinal*	*Paroaria*	*capitata*	Thraupidae
Yellow-fronted Canary*	*Crithagra*	*mozambica*	Fringillidae
Zebra Dove*	*Geopelia*	*striata*	Columbidae

Supplemental Figures

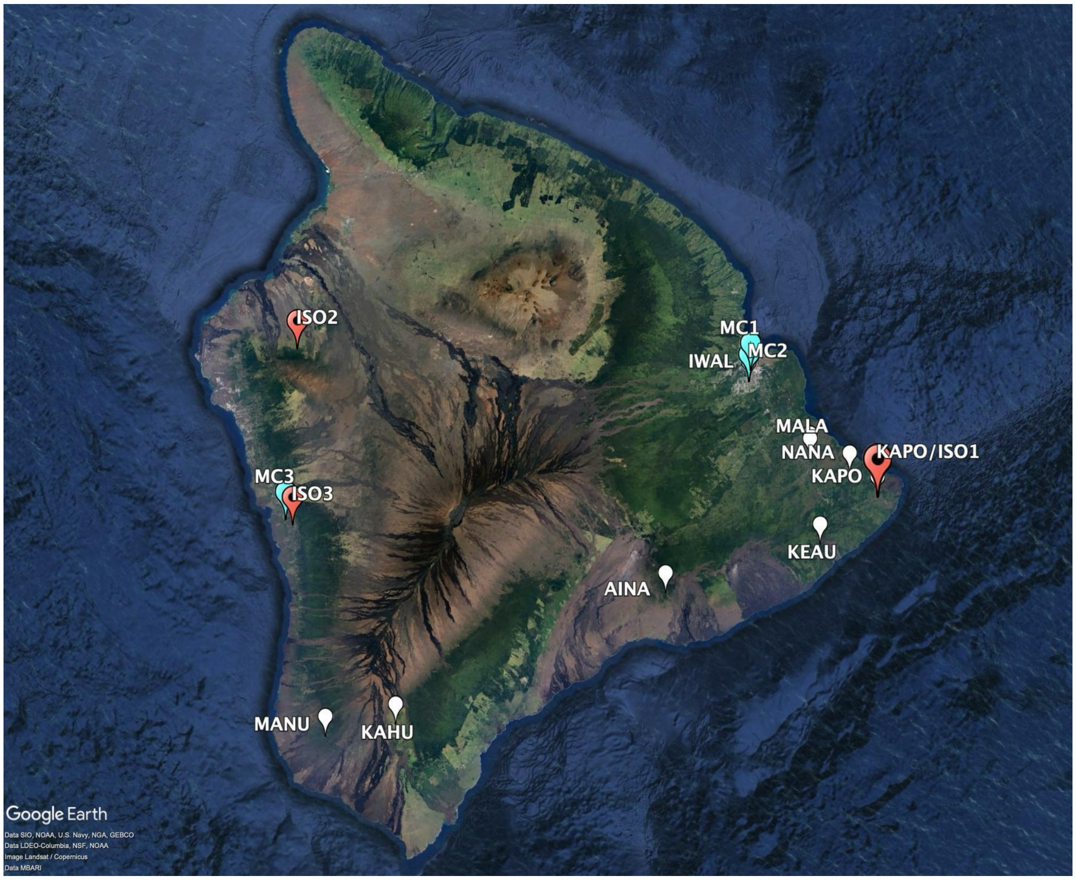

Figure S1. Locations of sites where bird communities were surveyed by point counts on the island of Hawai‘i. Red points indicate the origin of *Plasmodium relictum* (lineage GRW4) isolates used in experimental infection studies (ISO1, ISO2, ISO3). Blue points are collection locations for *Culex quinquefasciatus* egg rafts used for experimental infection studies (MC1: 19.7646407, -155.0924556°, elev. 55 m; MC2: 19.685787, -155.080147°, elev. 88 m; MC3: 19.4613276, -155.8981797, elev. 205 m). White points and one red point are point count sampling locations (described in Table S3) where we estimated bird relative abundance and community infectiousness. Note that additional sampling of birds occurred on several other islands to estimate parasitemias and relative infection prevalence (See Figure S9).

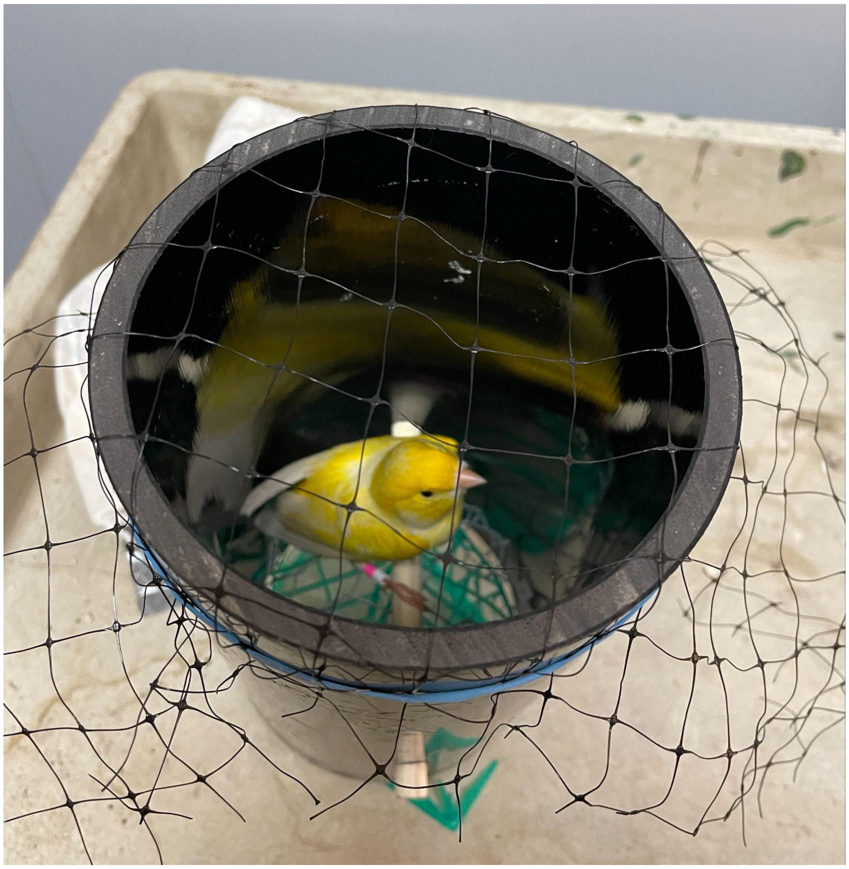

Figure S2. Mosquito feeding cylinder (10 cm diameter × 30 cm height) with an unrestrained domestic canary (*Serinus canaria*) on a wooden perch. The cylinder was elevated on a wire platform and capped with plastic mesh. This apparatus allowed mosquitoes to access birds from above and below but prevented birds from flying.

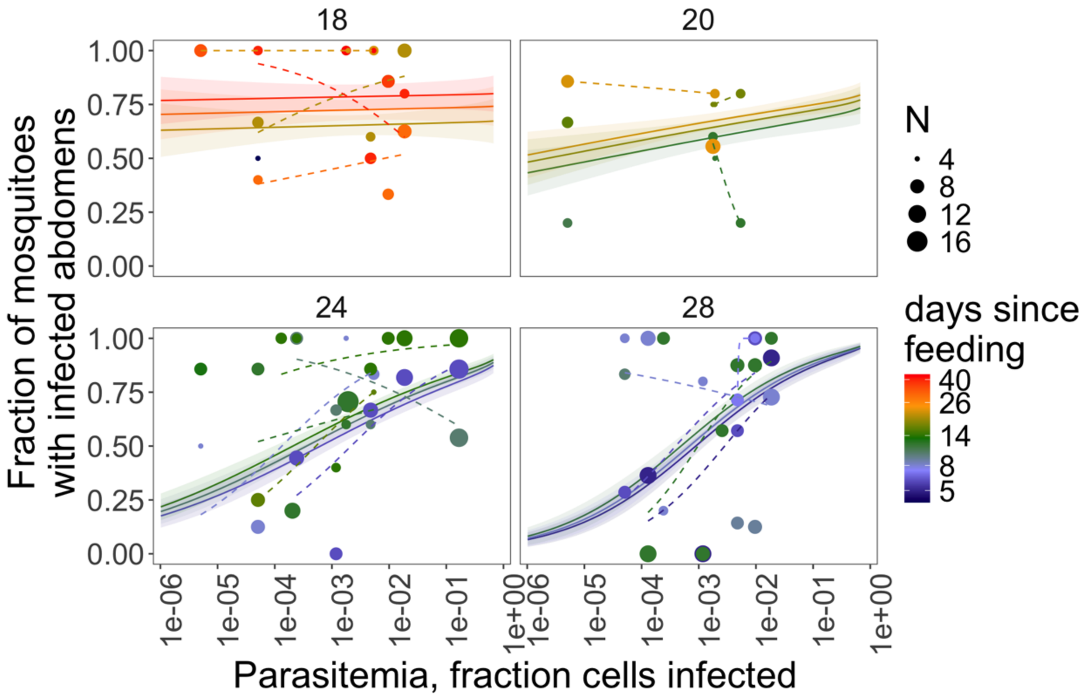

Figure S3. Fraction of mosquitoes with infected abdomens plotted against avian malaria parasitemia (fraction of red blood cells infected, on a $\log_{10}$ axis) for four temperatures (18, 20, 24, 28 °C) and a range of days since feeding. Points show groups of mosquitoes tested on a single day since feeding, with the size of points indicating sample size (mean 7, range 4-17), and color showing the number of days since feeding. Colored lines and ribbons show the fitted model with SE (Table S2). Dashed lines show individual fitted binomial regression lines to each set of points from the same day since feeding and temperature to illustrate the trends between parasitemia and the fraction of mosquitoes with infected abdomens.

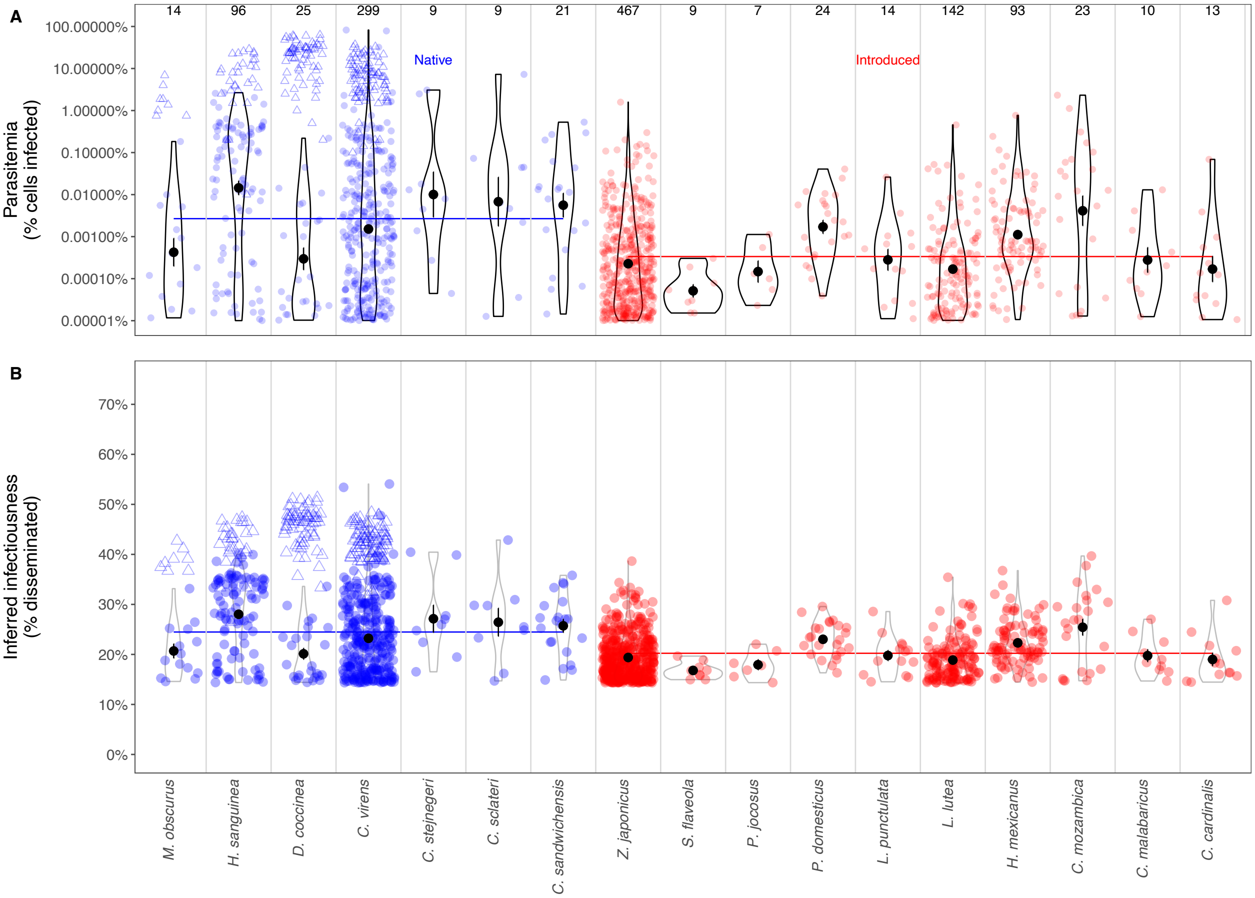

Figure S4. Parasitemia and inferred infectiousness, for seventeen bird species in Hawai‘i (7 native (blue), 10 introduced (red)). **A** *Plasmodium relictum* GRW4 parasitemia on a $\log_{10}$ scale. **B** Estimated fraction of mosquitoes with disseminated infections, 10 d at 24 °C after feeding on each parasitemia estimate from **A**. Points show values for individual wild-caught birds (circles) or samples from experimentally infected birds (triangles) during the first 90 d of infection taken from the literature. The number of parasitemia values for wild birds is displayed above each species (total N = 1,207). Black circles and error bars show the mean values ± SEM for each species for wild-caught birds (i.e., not including experimental infection data). Horizontal lines show the geometric mean of the average parasitemias **A** and mean infectiousness values **B** for native and introduced species to Hawai‘i (again, not including experimental infection data).

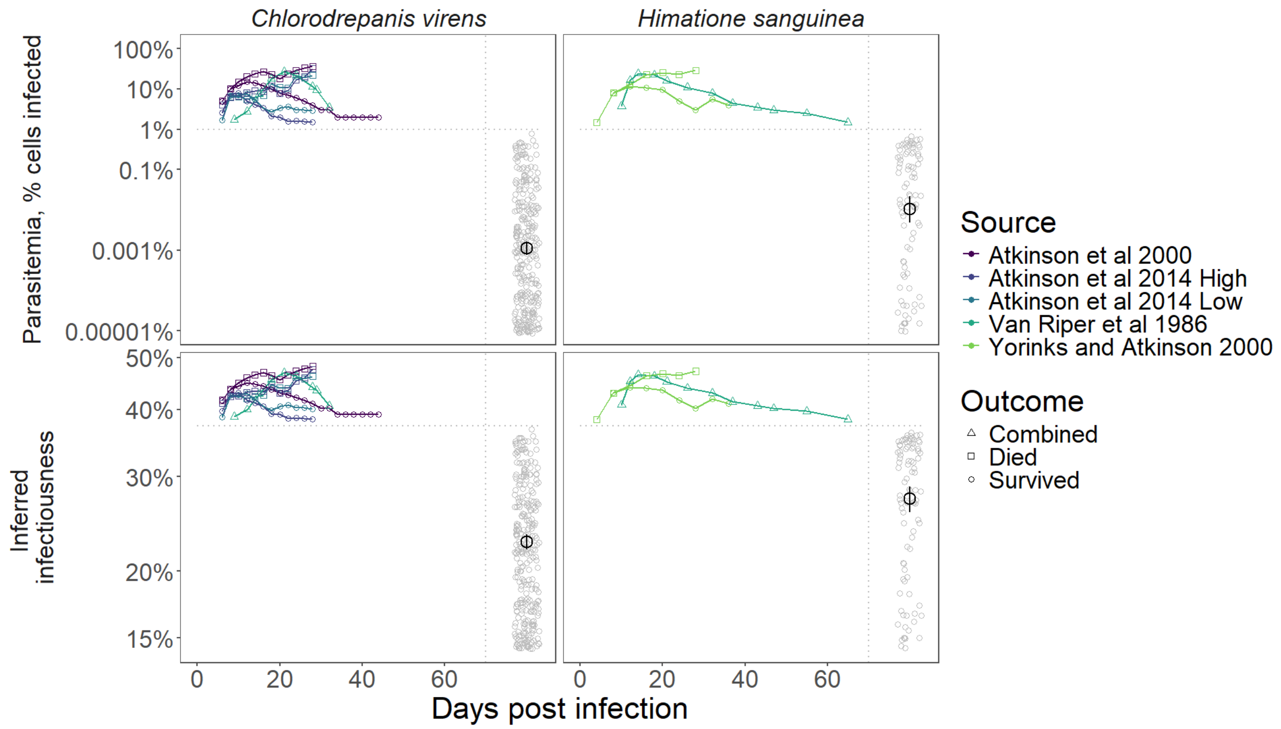

Figure S5. Parasitemia and inferred infectiousness for Hawai'i 'Amakihi (*Chlorodrepanis virens*) and 'Apapane (*Himatione sanguinea*) during the acute high parasitemia phase (colored points and lines in upper left of each panel), and chronic low parasitemia phase (gray points in the lower right of each panel). Black points show the geometric mean (parasitemia) or mean (Infectiousness) and 95% CI. High parasitemia data came from experimental infection studies[5–8] where 5-10 birds were sampled repeatedly (each point on each line is the mean of N = 5.1 (range 2-10) birds sampled on a given day). These studies sometimes separated data based on source population (high or low elevation) or survival outcome ("Combined" refers to studies that grouped data from both surviving and perishing birds), with birds that died having higher but shorter duration parasitemias than birds that survived. The low parasitemia data are from this study (Fig. 3; *C. virens* N = 290 and *H. sanguinea* N = 91).

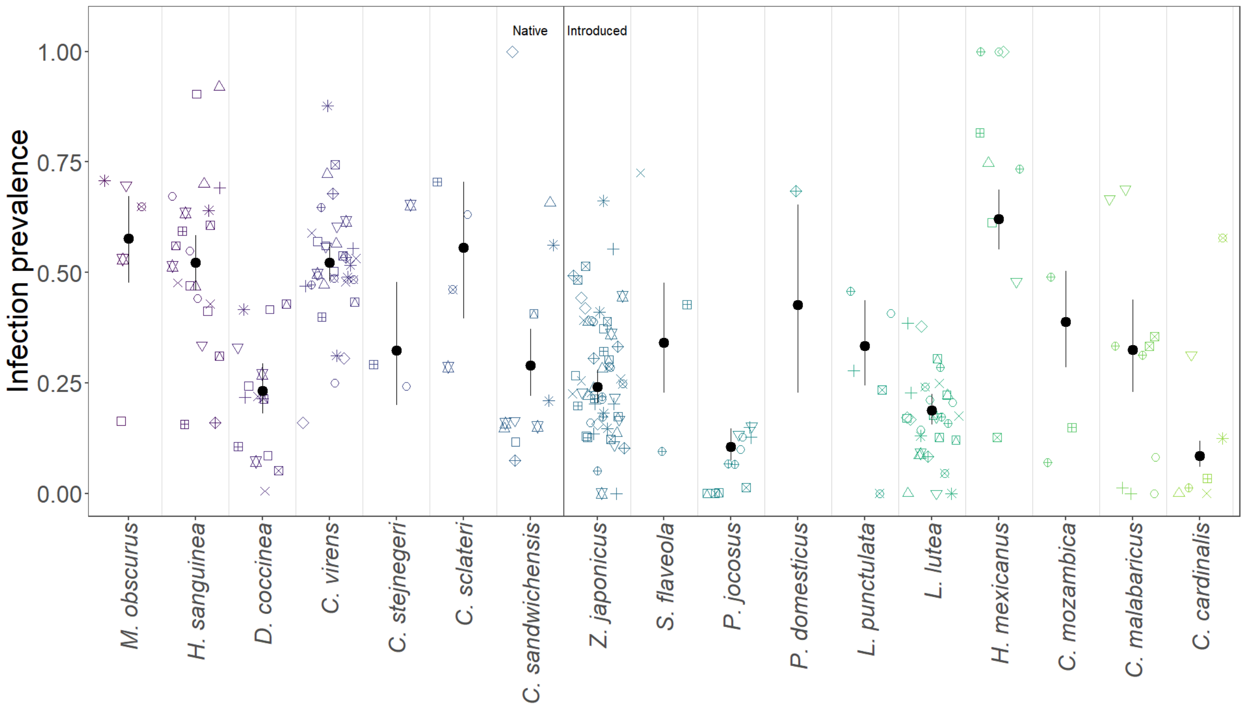

Figure S6. *Plasmodium relictum* infection prevalence by qPCR of 17 species of birds at 78 sites. Points show values from individual sites (indicated by point shape; no legend is given because there were 78 sites). Black points and error bars show predicted prevalence and SE for each species from a fitted model that included species, site, and age, for adult birds at an arbitrarily chosen moderate prevalence site (Pu‘u Wa‘awa‘a Forest Bird Sanctuary). Prevalence varied among species (χ^2=271.0, df = 32, P < 2.2 x 10^{-16}; only 17 shown in Figure) and sites (χ^2=613.3, df = 77, P < 2.2 x 10^{-16}), and age (χ^2=16.9, df = 3, P = 0.00073).

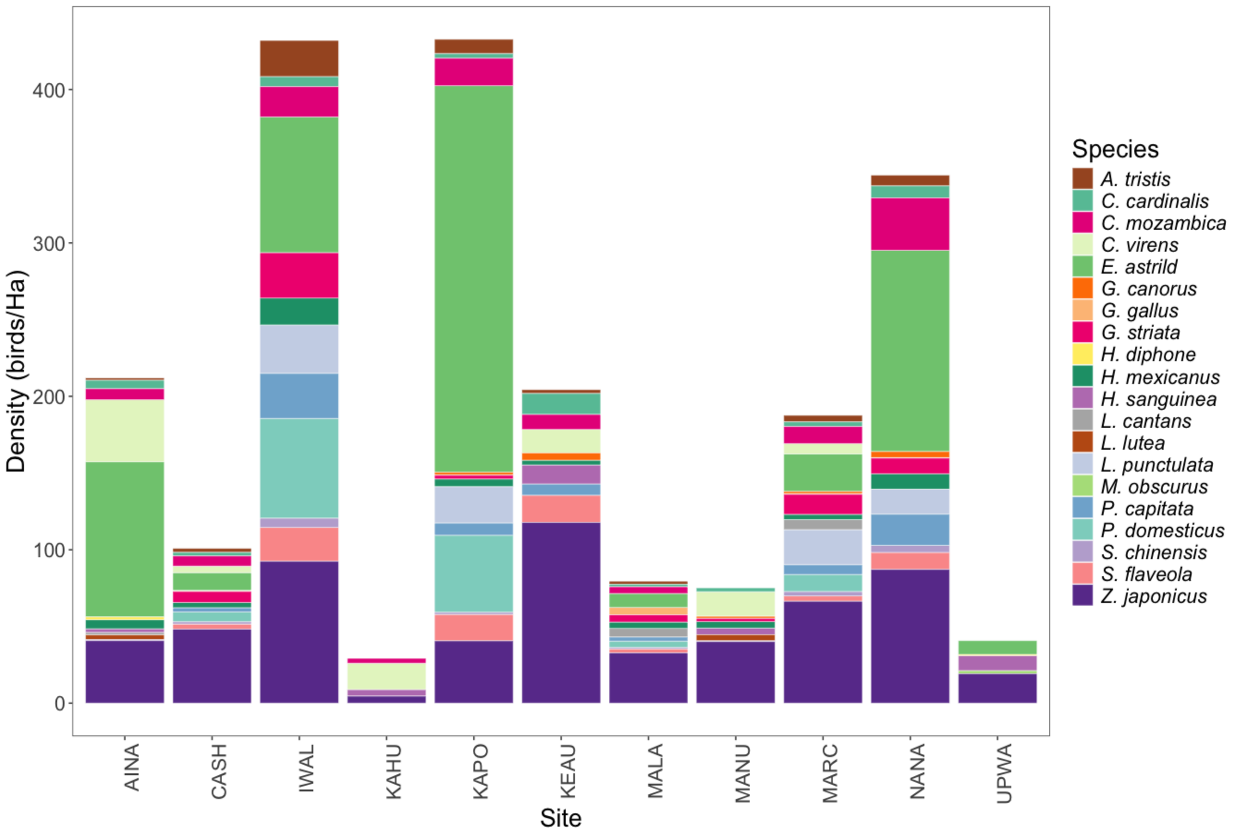

Figure S7. Density of birds at each of the 11 sites based on point counts and distance analysis. See Table S3 for site information.

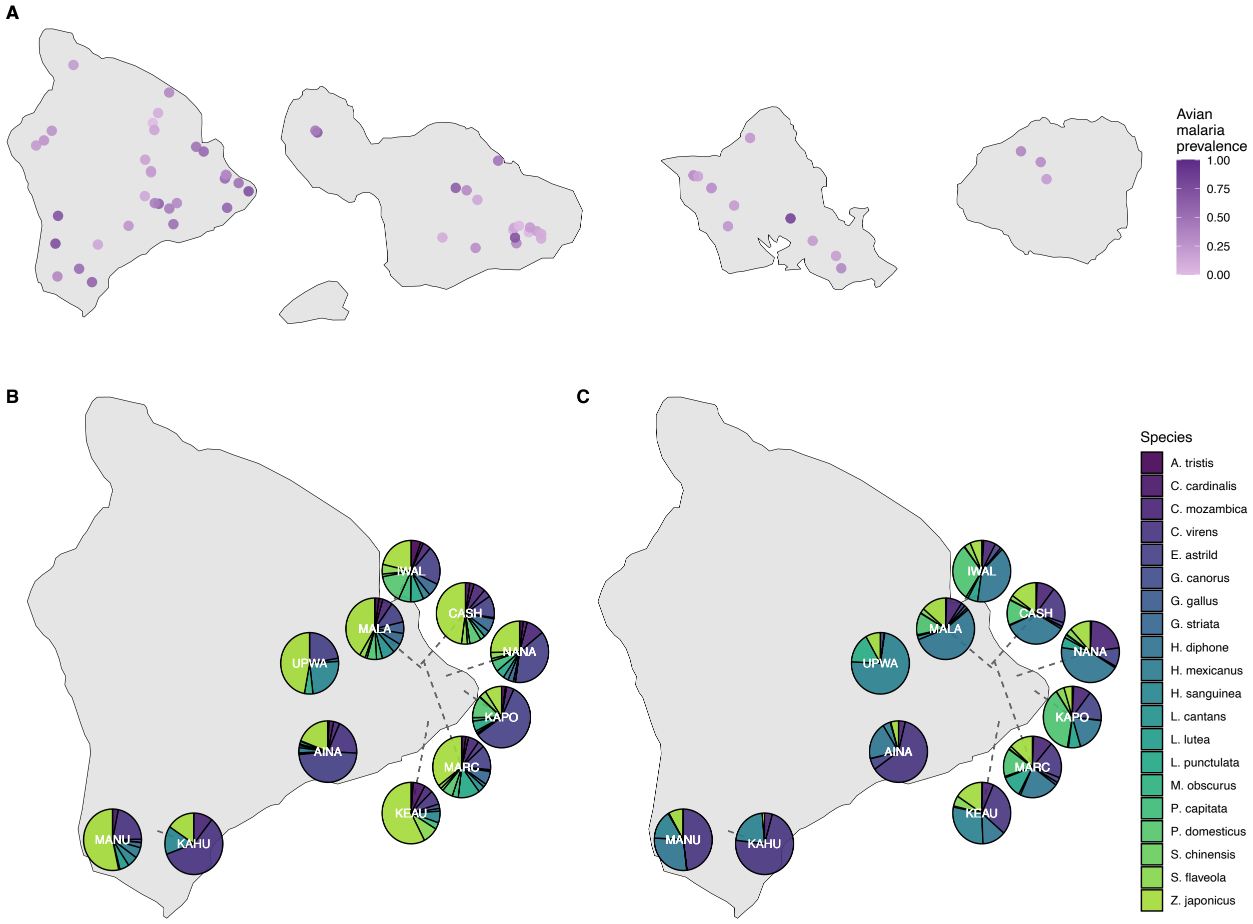

Figure S8. Sampled distribution of *Plasmodium relictum* in Hawai'i. **A** Infection prevalence in 64 sampled bird communities containing >8 individuals across 4 Hawaiian Islands. **B** Community composition and relative abundance of species at 11 sites on Hawai'i Island compared with **C** species' estimated contributions to transmission within the same sites.

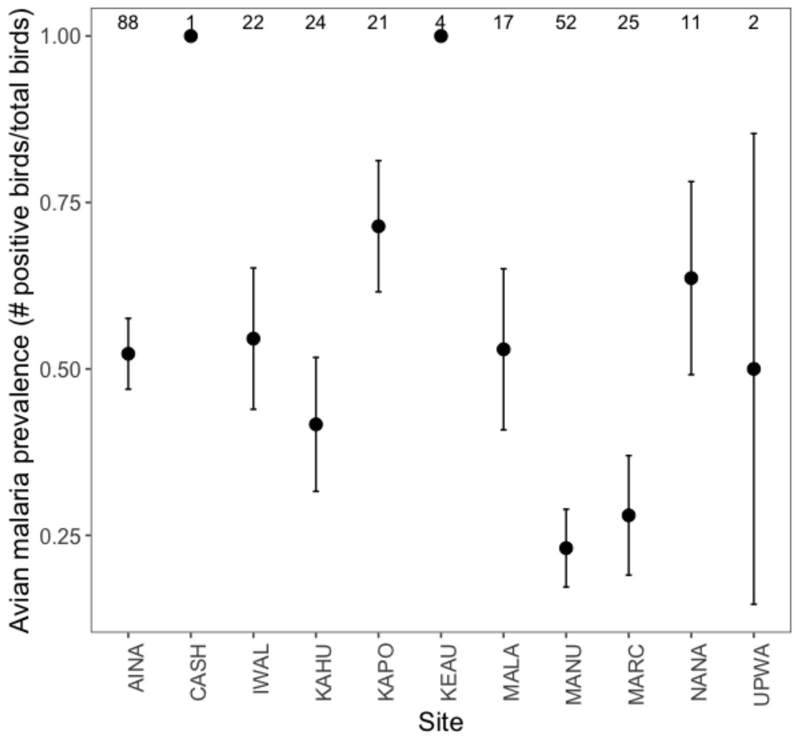

Figure S9. Prevalence (± SEM) of avian malaria infection at the 11 sites shown in Figure 4. Note that *Plasmodium relictum* was detected at all sites, despite small sample sizes at some sites. The number of birds tested is displayed above each site. See Table S3 for site information.

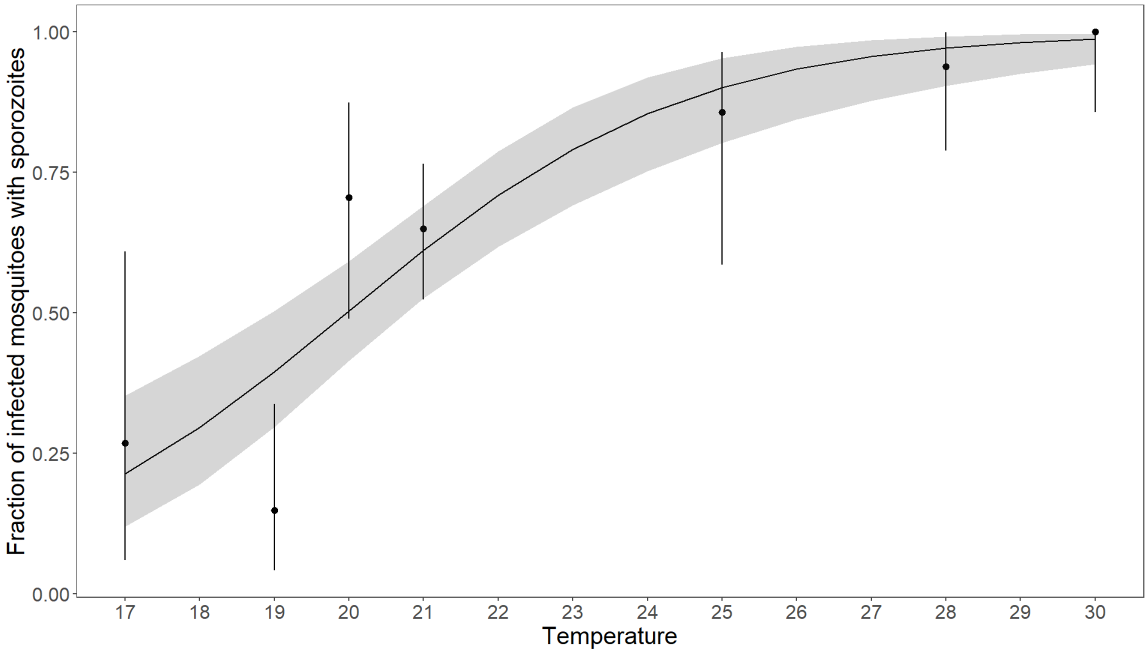

Figure S10. The fraction of mosquitoes with midgut oocysts that also had sporozoites in their salivary glands. Points show data from Lapointe et al. 2010[9] and the line shows a generalized linear model with a binomial distribution fit to the data. Error bars and ribbon show 95% confidence intervals for points and the fitted line, respectively.

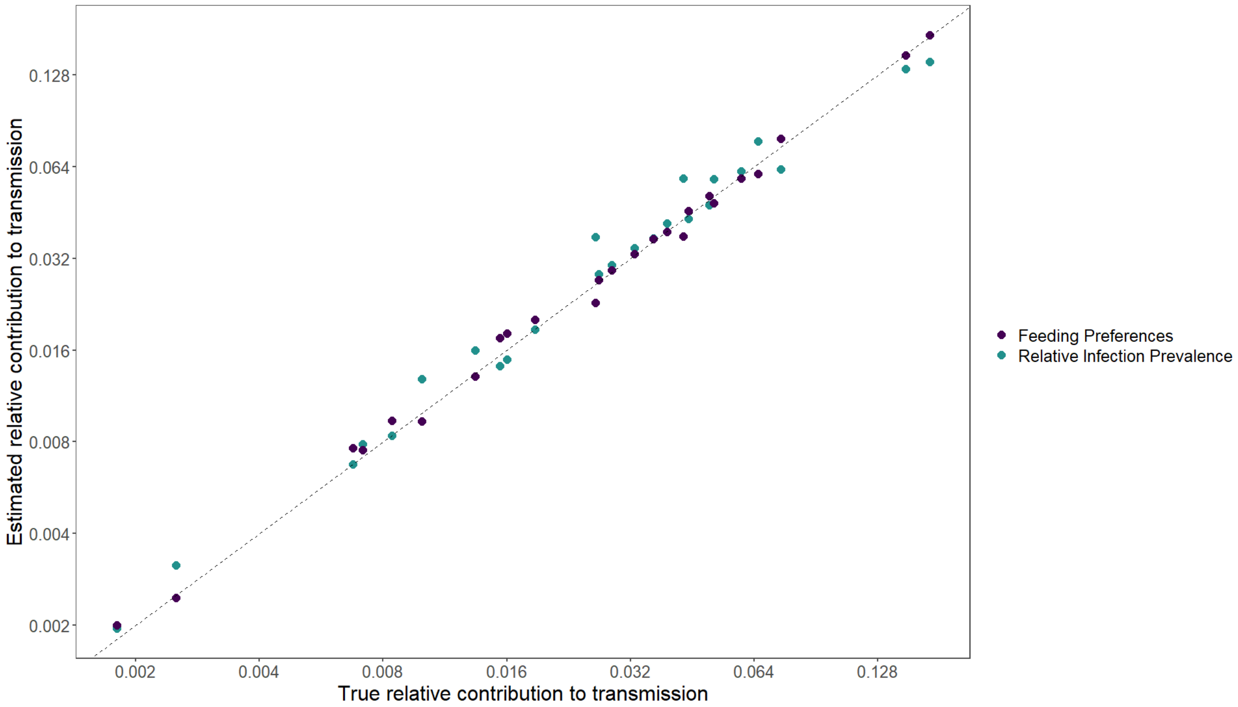

Figure S11. Results from model simulations comparing the accuracy of estimating the contribution of species to transmission using either mosquito relative host utilization measured directly via identifying the source of engorged mosquitoes and host abundance or host utilization estimated from relative infection prevalence when there is unmeasured variation in susceptibility and duration of detectable infection. Both axes are log-transformed, and the dashed line shows perfect agreement (1:1). Each of the 30 points is a simulated species.

Supplemental Information References

1. Brooks, M. *et al.* glmmTMB: balances speed and flexibility among packages for zero-inflated generalized linear mixed modeling. *The R Journal*, **9**, 378–400; doi:10.32614/RJ-2017-066 (2017).
2. R Core Team. R: A language and environment for statistical computing. R foundation for statistical computing (2022).
3. Hassan, H. K. *et al.* Avian host preference by vectors of eastern equine encephalomyelitis virus. *Am. J. Trop. Med. Hyg.* **69**, 641–647 (2003).
4. Kilpatrick, A. M., Daszak, P., Jones, M. J., Marra, P. P. & Kramer, L. D. Host heterogeneity dominates West Nile virus transmission. *Proc. R. Soc. B Biol. Sci.* **273**, 2327–2333 (2006).
5. Atkinson, C., Dusek, R., Woods, K. & Iko, W. Pathenogenicity of avian malaria in experimentally infected Hawaii amakihi. *J. Wildl. Dis.* **36**, 197–204 (2000).
6. Yorinks, N. & Atkinson, C. T. Effects of malaria on activity budgets of experimentally infected juvenile apapane (*Himatione sanguinea*). *The Auk* **117**, 731–738 (2000).
7. van Riper, C., van Riper, S., Goff, M. L. & Laird, M. The epizootiology and ecological significance of malaria in Hawaiian land birds. *Ecol. Monogr.* **56**, 327–344 (1986).
8. Atkinson, C. T., Saili, K. S., Utzurrum, R. B. & Jarvi, S. I. Experimental evidence for evolved tolerance to avian malaria in a wild population of low elevation Hawai‘i ‘amakihi (*Hemignathus virens*). *EcoHealth* **10**, 366–375 (2013).
9. LaPointe, D. A., Goff, M. L. & Atkinson, C. T. Thermal constraints to the sporogonic development and altitudinal distribution of avian malaria *Plasmodium relictum* in Hawai’i. *J. Parasitol.* **96**, 318–324 (2010).
