# Supplementary material for: Variation in pathogen load and the pathogen load–infectiousness relationship broaden avian malaria’s distribution
Source: Nat Commun. 2026 Feb 10;17:1213. doi: 10.1038/s41467-026-68927-x (PMC12891551; doi:10.1038/s41467-026-68927-x)
Supplement: Supplementary file 2 — Reporting Summary [file 41467_2026_68927_MOESM2_ESM.pdf]

nature portfolio

Corresponding author(s): Christa Seidl
Last updated by author(s): Jan 9, 2026

Reporting Summary

Nature Portfolio wishes to improve the reproducibility of the work that we publish. This form provides structure for consistency and transparency in reporting. For further information on Nature Portfolio policies, see our Editorial Policies and the Editorial Policy Checklist.

Statistics

For all statistical analyses, confirm that the following items are present in the figure legend, table legend, main text, or Methods section.

n/a	Confirmed	
☐	☒	The exact sample size (*n*) for each experimental group/condition, given as a discrete number and unit of measurement
☐	☒	A statement on whether measurements were taken from distinct samples or whether the same sample was measured repeatedly
☐	☒	The statistical test(s) used AND whether they are one- or two-sided<br>*Only common tests should be described solely by name; describe more complex techniques in the Methods section.*
☐	☒	A description of all covariates tested
☐	☒	A description of any assumptions or corrections, such as tests of normality and adjustment for multiple comparisons
☐	☒	A full description of the statistical parameters including central tendency (e.g. means) or other basic estimates (e.g. regression coefficient) AND variation (e.g. standard deviation) or associated estimates of uncertainty (e.g. confidence intervals)
☐	☒	For null hypothesis testing, the test statistic (e.g. *F*, *t*, *r*) with confidence intervals, effect sizes, degrees of freedom and *P* value noted<br>*Give P values as exact values whenever suitable.*
☒	☐	For Bayesian analysis, information on the choice of priors and Markov chain Monte Carlo settings
☒	☐	For hierarchical and complex designs, identification of the appropriate level for tests and full reporting of outcomes
☒	☐	Estimates of effect sizes (e.g. Cohen's *d*, Pearson's *r*), indicating how they were calculated

Our web collection on statistics for biologists contains articles on many of the points above.

Software and code

Policy information about availability of computer code

Data collection	No software was used to collect data.
Data analysis	All analyses were conducted in R (R version 4.4.1, http://www.r-project.org) using tidyverse (v2.0.0), glmmTMB (v1.1.11), lme4 (v1.1-35.5), brglm2 (v0.9.2), Distance (v2.0.1), ggplot2 (v3.5.2) and associated visualization packages (ggthemes, ggpubr, ggh4x, ggforce, viridis, RColorBrewer; all versioned), and spatial packages sf (v1.0-21) and rnaturalearth (v1.0.1). All software versions are reported on Dryad https://doi.org/10.5061/dryad.g1jwstr24

For manuscripts utilizing custom algorithms or software that are central to the research but not yet described in published literature, software must be made available to editors and reviewers. We strongly encourage code deposition in a community repository (e.g. GitHub). See the Nature Portfolio guidelines for submitting code & software for further information.

Data

Policy information about availability of data

All manuscripts must include a data availability statement. This statement should provide the following information, where applicable:

- Accession codes, unique identifiers, or web links for publicly available datasets
- A description of any restrictions on data availability
- For clinical datasets or third party data, please ensure that the statement adheres to our policy

The datasets and code generated and/or analyzed during the current study are available on Dryad at https://doi.org/10.5061/dryad.g1jwstr24

Research involving human participants, their data, or biological material

Policy information about studies with human participants or human data. See also policy information about sex, gender (identity/presentation), and sexual orientation and race, ethnicity and racism.

Reporting on sex and gender	N/A
Reporting on race, ethnicity, or other socially relevant groupings	N/A
Population characteristics	N/A
Recruitment	N/A
Ethics oversight	N/A

Note that full information on the approval of the study protocol must also be provided in the manuscript.

Field-specific reporting

Please select the one below that is the best fit for your research. If you are not sure, read the appropriate sections before making your selection.

☐ Life sciences ☐ Behavioural & social sciences ☒ Ecological, evolutionary & environmental sciences

For a reference copy of the document with all sections, see nature.com/documents/nr-reporting-summary-flat.pdf

Ecological, evolutionary & environmental sciences study design

All studies must disclose on these points even when the disclosure is negative.

Study description

We combined field surveillance and experimental infections to examine avian malaria transmission dynamics in Hawaiʻi. To experimentally quantify the relationship between parasitemia and mosquito infectiousness, we fed Culex quinquefasciatus on domestic canaries (Serinus canaria) with 21 parasitemia levels between 0.0050–59.4%. Mosquitoes were held at three constant temperatures (20 °C, 24 °C, and 28 °C) and sampled at multiple days post-feeding. The study followed a factorial design with treatment factors including parasitemia, temperature, and days post-feeding, and their interactions. Each bird–mosquito feeding event represented an experimental unit. Infection status was determined by qPCR of mosquito abdomens (midgut infection) and pooled thorax/head/legs (disseminated infection), providing multiple biological replicates for each treatment combination. To get wild bird infection data, we mist-netted across 78 sites and tested blood samples for Plasmodium relictum infection by qPCR. We described avian community composition using auditory surveys at 11 of our 78 sites.

Research sample

Field sampling and collection:
We collected blood samples from 4,218 individuals from 34 bird species captured at 78 sites across four Hawaiian Islands (Hawaii, Maui, Oahu, Kauai). These sites spanned a range of elevations and habitats and were chosen to represent the diversity of bird community compositions, Plasmodium relictum parasitemias and prevalences. Passerines (Order Passeriformes) were the primary wild bird taxa sampled, encompassing both native Hawaiian forest birds and introduced species.

Culex quinquefasciatus mosquitoes, reared from egg rafts collected from 3 locations on Hawaiʻi Island (detailed in ms), were used as vectors in controlled feeding experiments to represent the primary natural mosquito vector in Hawaiʻi. Mosquitoes were exposed to infected canaries under four constant temperatures and sampled at multiple time points to capture variation in infection, dissemination, and infectiousness. Up to 100 mosquitoes were exposed to birds each feeding night in order to ensure the maximum number of blood feeding events under semi-natural (unrestrained bird, nocturnal feeding) as possible and deemed safe for bird blood loss. We were able to generate 820 blood-fed mosquitoes.

The three P. relictum GRW4 isolates (detailed in ms) used in the bird and mosquito experimental infections were originally collected in blood samples from 3 separate wild birds representing two different avian communities in Hawaii (low-elevation exotic, middle elevation mixed native and exotic). Note, past studies found limited genetic variation and no difference in pathogenicity between Hawaiian isolates of GRW4. Our isolates were preserved as individual isolate lines throughout the study. Individual birds were only ever infected with one isolate; previous studies indicate there is no difference in the pathogenicity of Hawaiian isolate strains.

Laboratory infections:
Domestic canaries (Serinus canaria, N=45), sourced from local breeders in Hawaiʻi and California and free of prior malaria infection, were experimentally inoculated with P. relictum to cycle and preserve P. relictum isolates for experimental infection studies, and when exposed to mosquitoes, used to achieve a broad range of parasitemia levels. This ensured coverage of infection intensities relevant to natural wild-bird infections. When possible and permitted, multiple parasitemia feeding trials occurred with the same individuals. For our assessment of the role the acute and chronic infections stage of infection play in transmission, we incorporated experimental infection data in Hawaiian birds from previously published literature. These are all cited appropriately in the ms.

Sampling strategy

We maximized sample sizes within the logistical and ethical constraints of the study. Wild birds were captured under state and

Sampling strategy

federal permits, and capture numbers varied across passive mist-netting sessions due to natural fluctuations in bird activity and abundance. To ensure robust prevalence estimates while retaining the majority of collected data (>80%), we restricted avian malaria prevalence analyses to bird communities with more than eight individuals sampled. For mosquito feeding trials, we exposed as many mosquitoes to infected birds as possible, given the concurrent availability of mosquitoes and limits on blood collection set by the attending veterinarian and IACUC to ensure bird welfare. We included data from all blood fed mosquitoes. We aimed to replicate feeding events across a range of parasitemias, but replication was inherently constrained by the natural variability in infection dynamics among and within individual birds and vectors.

Data collection

Wild Bird Capture and Blood Sampling:
Wild birds were captured across 78 forested sites ranging from 29 to 2,000 meters in elevation on the Hawaiian Islands of Kaua'i, O'ahu, Maui, and Hawai'i between 2015 and 2022. Birds were captured using 38-mm mesh mist nets. Upon capture, each bird was identified to species, and blood samples of 25–100 µL were collected by brachial venipuncture. The blood was immediately placed into 1 mL of Queen's Lysis Buffer to preserve DNA. Samples were stored either at ambient temperature for up to 90 days or frozen at -20 °C prior to molecular analysis. Captures and sample processing were preformed by authors CMS, AMK and EA, JTF, EHP with their respective field teams. These samples were used to quantify Plasmodium relictum infection prevalence and parasitemia by quantitative PCR (qPCR).

Plasmodium Collection and Bird Infection:
Three isolates of Plasmodium relictum (lineage GRW4) were obtained from wild-caught birds using similar protocols to those previously described to capture and blood sample from wild birds. Up to 100 microliters of bird blood from each isolate was screened for infection and if positive, inoculated intramuscularly into an uninfected canary. Freshly collected infected blood was cycled 1-5 times in canaries to ensure parasite viability and maximize infection doses prior to mosquito feeding trials or cryopreservation. All procedures involving wild or laboratory birds complied with Institutional Animal Care and Use Committee protocols approved at the University of California, Santa Cruz (protocol Kilpm2003).
For experimental infections, naïve canaries were inoculated intramuscularly with 50–200 µL of infected whole blood containing P. relictum at parasitemias ranging from 0.1% to 4.25%. Infected blood used for inoculation was either fresh (from previously passaged canaries) or thawed from cryopreserved stocks. Starting five days post-inoculation, blood was sampled every three days by brachial venipuncture (5–10 µL per sampling) to monitor infection status and parasitemia using thin blood smears and quantitative PCR (qPCR). Once parasitemia became detectable, infected canaries were used as hosts in mosquito feeding experiments. CMS, II, and AMK conducted all experimental infections of birds.

Mosquito Collection, Rearing, and Experimental Infection Assays:
Egg rafts of Culex quinquefasciatus were collected from three sites on Hawai'i Island from 2020 to 2023 by author CMS. The egg rafts were transported within 24 hours to the laboratory in Santa Cruz, California, where larvae were hatched and reared under controlled conditions (26 °C, 70–80% humidity, 12:12 light:dark cycle). Larvae were fed ground fish food daily until pupation, and adults emerged into cages where they were provided ad libitum 10% sucrose solution. Mosquitoes aged 5 to 37 days (mean 20 days) from wild-caught and first- and second-generation lab-reared cohorts were starved of sucrose for 48 hours prior to feeding experiments. For infection assays, canaries experimentally infected with P. relictum were placed inside mosquito cages overnight (10 hours) to allow mosquitoes to blood-feed. Engorged mosquitoes were then incubated at four temperature treatments (18, 20, 24, and 28 °C) and dissected at predetermined time points to assess infection progression. Authors CMS, II, SL, DL and RM were responsible for mosquito and bird care and mosquito dissection.

Mosquito Dissection and qPCR Detection of Infection:
Dissections were performed by carefully separating mosquito abdomens from the thorax, head, and legs under sterile conditions. Abdomen and combined thorax/head/legs samples were stored separately in 70% ethanol and frozen at -20 °C until DNA extraction. A bead-beating step was used during extraction to ensure thorough tissue disruption. Quantitative PCR targeted the Plasmodium relictum cytochrome b gene specific to the GRW4 lineage, allowing sensitive detection and quantification of parasite DNA in mosquito tissues. Mosquito infection was classified as abdomen infection or disseminated infection (presence of parasite DNA in thorax/head/legs), the latter serving as a proxy for mosquito infectiousness. Authors KLP and KB established protocols for and conducted all DNA processing and qPCR.

Auditory Surveys and Estimation of Bird Community Composition:
At 11 forested sites on Hawai'i Island, bird communities were characterized using unlimited-distance point counts. Each site had four point counts, each lasting six minutes, conducted between 0600 and 1000 hours during the breeding season (February to June 2020). Point count locations were spaced at least 200 meters apart to avoid double-counting. Bird detections were recorded with distances estimated to each individual. CMS conducted all counts.

Timing and spatial scale

The temporal and spatial scope was selected to balance logistical feasibility with capturing natural variation in bird communities, parasite prevalence, and mosquito infection dynamics. This study was conducted over multiple years. Bird sampling occurred between 2015 and 2023 to make use of the largest dataset tested by qPCR to date. Wild bird blood samples were collected across four Hawaiian Islands—Kaua'i, O'ahu, Maui, and Hawai'i Island—spanning elevations from 29 to 2,000 meters. The spatial breadth of these sites was chosen to encompass diverse bird communities and environmental conditions where avian malaria transmission occurs. Sampling frequency varied by site and year, but aimed to capture broad temporal representation within and across years.
At community census sites on Hawai'i Island, auditory surveys were conducted from February to June 2020, with four 6-minute point counts per site conducted between 0600 and 1000 hours, separated by at least 200 meters. This timing targeted peak bird activity during morning hours and the breeding season, maximizing detectability and abundance estimates.
Mosquito egg rafts and avian malaria isolates were collected from three locations on Hawai'i Island between 2020 and 2023. Mosquitoes were reared for experimental infections. Laboratory mosquito feeding assays occurred, as birds and mosquito availability made possible, between these collection years. Mosquito blood feedings and subsequent dissections were performed at multiple time points up to 44 days post-feeding to capture the known temperature-dependent development of disseminated infections in mosquitoes. Temperatures chosen for mosquito incubation (18, 20, 24, and 28 °C) reflect the range of temperatures common in Hawaiian bird habitats.

Data exclusions

We analyzed all data.

Reproducibility	For each mosquito feeding trial, we attempted to repeat experiments under similar biological conditions, specifically using birds with comparable parasitemia levels and mosquito cohorts of the same age range and origin. These attempts were not always successful, as we were not able to control bird parasitemia or guarantee mosquito feeding success. However, given our ability to expose and infect birds and mosquitoes multiple times across different individuals, years, and with different staff, we've help demonstrate reproducibility. All blood samples were tested in duplicate or triplicate to test the reproducibility of results in the same lab and all protocols for our qPCR are currently published.
Randomization	Wild birds were captured using standardized, passive mist-netting protocols at multiple sites, and capture order was determined by the timing of incidental encounters rather than any pre-selection, resulting in de facto random sampling from the available community. Birds were allocated to mosquito feeding trials based on the presence of Plasmodium infection, as determined by malaria screening. When multiple infected birds were available, individuals were selected in random order for trials. Mosquitoes used in feeding trials were drawn randomly from cages arising from the same egg raft. All mosquito feedings were conducted under consistent environmental conditions (temperature, light cycle, and rearing protocols). Individual female mosquitoes were only exposed one time to an infected bird to account for bias in infection results due to multiple feedings. Only females that appeared visibly blood fed (i.e., with a distended, red abdomen) were monitored and dissected.
Blinding	N/A

Did the study involve field work? ☒ Yes ☐ No

Field work, collection and transport

Field conditions	Our sites span the majority of possible forest types between 29 to 2,000 meters on the islands of Hawai'i, Oahu, Maui and Kauai. Some are characterized as suburban, agricultural, wet/dry native Metrosideros polymorpha forest, wet/dry non-native forest, or bog/wetland. While not measured as part of this study, rainfall and temperature at these sites is known to vary. Temperatures in Hawai'i generally decrease with elevation, with average annual temperatures ranging from about 24–26 °C near sea level (around 20 m) to roughly 10–15 °C at 2,000 m elevation. Rainfall is often highest on windward facing slopes at middle to high elevations (2,000-4,000 mm per year).
Location	As we sampled from >78 sites, these are detailed extensively with locations (lat/long, elevation) and forest types in the submitted Supplemental Material (See Figure S1, Table S3).
Access & import/export	We conducted fieldwork under Hawai'i Division of Forestry and Wildlife Protected Wildlife Permits (WL19-23; WL 17-08), USGS Bird Banding Laboratory permit numbers (#23600, #21144), and a Hawai'i State Access and Forest Reserve Special Use Permit. We collected mosquito egg rafts with U.S. Veterinary Permits 139503 and 611-21-327-00722.
Disturbance	Disturbance to Birds:<br>Our study involved capturing wild birds using mist nets and collecting small blood samples via brachial venipuncture. Although minimally invasive, these activities can cause short-term stress to the birds and potential minor injury. To minimize impacts, all bird handling was conducted by trained personnel following established ethical protocols and with appropriate permits. Blood volumes drawn were limited to amounts deemed safe by veterinary and IACUC guidelines, minimizing harm or long-term effects on the birds. Birds were released promptly at the site of capture after sampling. Sampling was limited to ~1 week at any one site.<br><br>Disturbance to Landscape:<br>Bird capture and point count surveys took place in forested habitats across multiple Hawaiian Islands. Mist net setup and auditory surveys involved minimal physical disturbance, with nets installed carefully to avoid damage to vegetation. Fieldwork was planned to avoid sensitive breeding periods and high visitor traffic to reduce additional stress on wildlife and minimize habitat disturbance.

Reporting for specific materials, systems and methods

We require information from authors about some types of materials, experimental systems and methods used in many studies. Here, indicate whether each material, system or method listed is relevant to your study. If you are not sure if a list item applies to your research, read the appropriate section before selecting a response.

Materials & experimental systems

n/a	Involved in the study	
☒	☐	Antibodies
☒	☐	Eukaryotic cell lines
☒	☐	Palaeontology and archaeology
☐	☒	Animals and other organisms
☒	☐	Clinical data
☒	☐	Dual use research of concern
☒	☐	Plants

Methods

n/a	Involved in the study	
☒	☐	ChIP-seq
☒	☐	Flow cytometry
☒	☐	MRI-based neuroimaging

Animals and other research organisms

Policy information about studies involving animals; ARRIVE guidelines recommended for reporting animal research, and Sex and Gender in Research

Laboratory animals	Domestic canary, Serinus canaria, aged 1-2 years
Wild animals	We collected blood samples from 4,218 individuals from 34 bird species found on the Hawaiian Islands using mist-nets. All birds were released at site of capture within 1 hr. A complete list of the species sampled is provided in the Supplemental Material Table S5.
Reporting on sex	Sex was not quantified as a factor during this study. Sex-based testing was not conducted on blood samples and for many species, the sexes are not easily visually distinguished.
Field-collected samples	Blood samples collected from wild birds were immediately preserved in 1 mL of Queen's Lysis Buffer and stored at room temperature for up to 90 days or frozen at -20 °C for longer-term storage before DNA extraction. Isolate samples were cryopreserved in glycerolyte solution and are stored in -80 °C. Mosquito samples were separated into abdomen and combined head/thorax/legs groups and preserved in 70% ethanol. These samples were stored frozen at -20 °C for periods ranging from 1 to 90 days prior to DNA extraction and qPCR analysis. All samples were either destroyed in analysis or are currently in freezer storage at UC-Santa Cruz with author AMK.
Ethics oversight	Our work with was reviewed and approved by the Institutional Animal Care and Use Committee at the University of California in Santa Cruz, USA (Kilpm2003) and bird banding/capture permits were approved by the USGS Bird Banding Laboratory (permit 23600 & 21144) , which sets high standards for permitting and reporting with wild animals.

Note that full information on the approval of the study protocol must also be provided in the manuscript.

Plants

Seed stocks	N/A
Novel plant genotypes	N/A
Authentication	N/A
